# In Silico Evaluation via Virtual Screening, ADMET Profiling and Molecular Dynamics Simulations: Synthesis and Biological Activity Analysis of a Novel Antitumor PI3K Inhibitor

**DOI:** 10.3390/biology15191742

**Published:** 2026-10-01

**Authors:** Wenqing Jia, Yukui Ma, Zhe Zhang

**Affiliations:** 1 School of Chemistry and Chemical Engineering, Qilu Normal University, Jinan 250200, China; 2 Tianjin Key Laboratory of Technologies Enabling Development of Clinical Therapeutics and Diagnostics, School of Pharmacy, Tianjin Medical University, Tianjin 300070, China

**Keywords:** KTC101, PI3K inhibitor, tumor

## Abstract

The PI3K plays a crucial role in cancer progression, and the development of PI3K inhibitors has always been a hot research direction in oncology. Through virtual screening and docking, potential lead compounds were identified. ADMET prediction revealed that KTC101 possesses superior pharmacokinetic profile. Molecular dynamics simulations indicated that compound KTC101 could stably bind to PI3Kδ, with a binding free energy of −374.627 kJ/mol. Analysis of the metabolism of KTC101 revealed that this compound could undergo diverse phase I metabolic reactions catalyzed by hepatic CYP450 enzymes to form highly polar metabolites, indicating its strong metabolic potential and easy metabolic clearance in vivo. Then it was synthesized and evaluated for kinase inhibitory activity and antiproliferative effects in HNSCC cell lines. KTC101 was first reported as a PI3K inhibitor, with IC_50_ values of 191.23 ± 40.84 nM, 23.30 ± 5.68 nM, and 146.20 ± 21.82 nM for PI3Kα, PI3Kδ and PI3Kγ, respectively, and demonstrated dose-dependent inhibition of cancer cell growth in vitro, with IC_50_ values of 4.1 ± 0.73 μM for HSC3 cells and 2.2 ± 0.11 μM for HSC4 cells. It exhibited inhibitory activity against PI3K, showing greater inhibitory potency toward PI3Kδ. This study demonstrates the anti-tumor potential of KTC101.

## Simple Summary

Certain natural proteins inside human cells can speed up the growth and spread of cancer, and creating new medicines to block these proteins is a major focus of cancer research. This study set out to discover and test a new potential anticancer molecule to block one such cancer-driving protein. Researchers first used computer modeling to pick out this promising molecule, confirmed it could attach firmly to its target protein, and verified that the liver could easily break this molecule down and clear it from the body after use. The team then made this molecule in the lab and ran tests to measure its effects. Laboratory tests showed this newly identified molecule effectively blocks the problematic protein, with its strongest blocking effect on one specific form of the protein, and it slows the growth of two different types of head and neck cancer cells grown at low doses. This work confirms the molecule is a viable candidate for further development into new cancer treatments, which may offer new treatment choices for people living with head and neck cancer in the future.

## 1. Introduction

**Figure 1:**
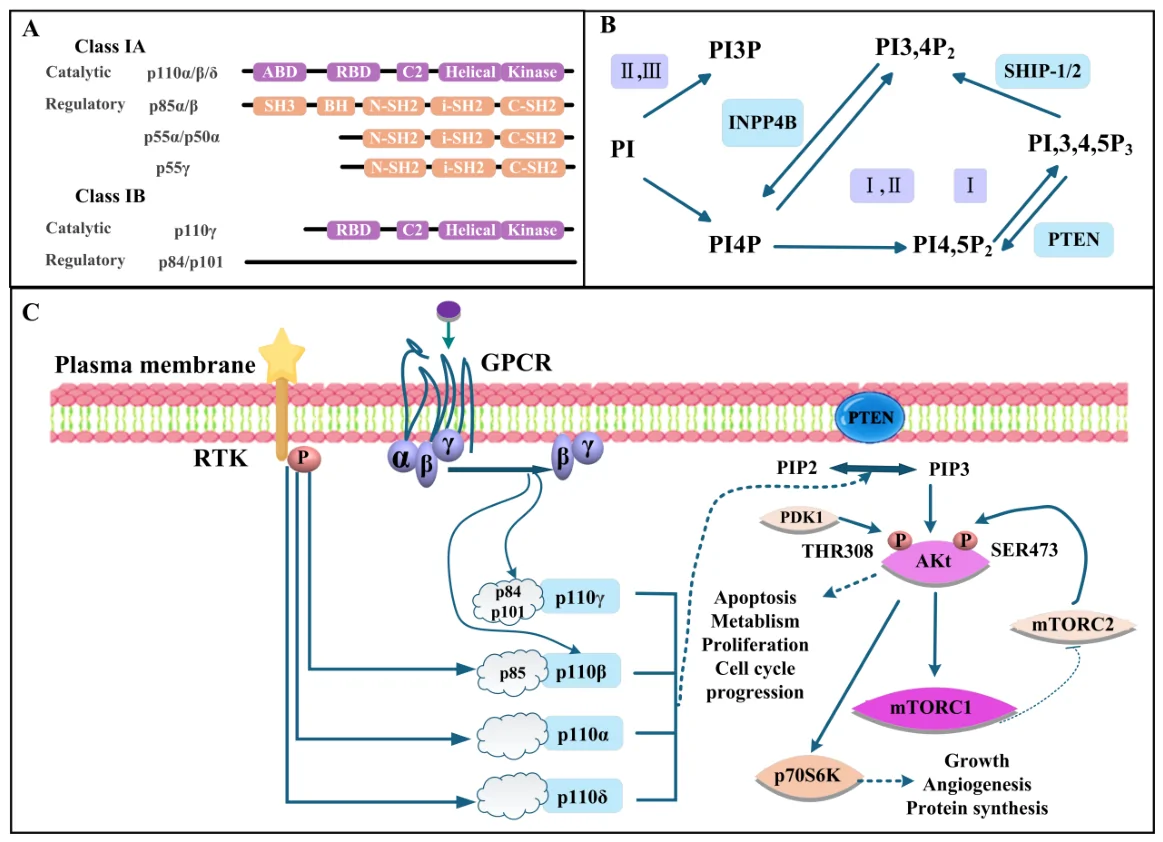
(**A**) Structure of class I PI3Ks. (**B**) Schematic diagram for synthesis and metabolism of PtdIns polyphosphates. (**C**) The PI3K/Akt/mTOR signaling pathway. Abbreviations: ABD, adaptor binding domain; RBD, ras binding domain; SH3, Src homology 3; BH, BCR-homology; N-SH2/i-SH2/C-SH2, N-terminal/intermediate/C-terminal Src-homology 2; PI, phosphatidylinositol; PI3P, phosphatidylinositol 3-phosphate; PI4P, phosphatidylinositol 4-phosphate; PI4,5P_2_, phosphatidylinositol 4,5-bisphosphate; PI3,4P_2_, phosphatidylinositol 3,4-bisphosphate; PI3,4,5P_3_, phosphatidylinositol 3,4,5-trisphosphate; PIP2, phosphatidylinositol 4,5-bisphosphate; PIP3, phosphatidylinositol 3,4,5-trisphosphate; INPP4B, inositol polyphosphate 4-phosphatase type II; SHIP, SH2-containing inositol 5-phosphatase; PTEN, phosphatase and tensin homolog; RTK, receptor tyrosine kinase; GPCR, G protein-coupled receptor; PDK1, 3-phosphoinositide-dependent protein kinase 1; mTORC1/2, mechanistic target of rapamycin complex 1/2; p70S6K, p70 ribosomal S6 kinase.

Phosphatidylinositol 3-kinase (PI3K) is a key hub connecting intracellular and extracellular signal transduction, modulating multiple biological activities such as cell growth, proliferation, survival, migration, and metabolism [1,2]. PI3K is categorized into three subfamilies: classes I, II, and III [2,3]. As shown in Figure 1A, class I PI3Ks are heterodimers composed of a catalytic subunit (p110α/β/δ for class IA and p110γ for class IB) and a regulatory subunit (p85α/β, p55α/p50α or p55γ for class IA; p84/p101 for class IB). The catalytic subunit contains an N-terminal adaptor binding domain (ABD), a Ras binding domain (RBD), a C2 domain, a helical domain, and a kinase domain, whereas the regulatory subunit contains an Src homology 3 (SH3) domain, a BCR-homology (BH) domain, two Src homology 2 (SH2) domains (nSH2 and cSH2), and an inter-Src homology 2 (iSH2) domain [3,4]. Existing evidence indicates that class I PI3K is tightly associated with the progression of multiple human malignancies, including head and neck squamous cell carcinoma (HNSCC), breast cancer, and ovarian cancer, and this class consists of four isoforms: PI3Kα, PI3Kβ, PI3Kδ and PI3Kγ [4,5]. Of these isoforms, PI3Kα and PI3Kβ are widely distributed in most tissue cells, whereas PI3Kδ and PI3Kγ are predominantly expressed in immune cells [6,7,8].

The PI3K/Akt/mTOR cascade is one of the most canonical signaling axes involving PI3K and is tightly implicated in tumor initiation and progression [9,10]. As illustrated in Figure 1B, phosphatidylinositol (PI) is sequentially phosphorylated to phosphatidylinositol 3-phosphate (PI3P), phosphatidylinositol 4-phosphate (PI4P) and phosphatidylinositol 4,5-bisphosphate (PI4,5P_2_, PIP2), which is further converted to phosphatidylinositol 3,4,5-trisphosphate (PI3,4,5P_3_, PIP3) by class I PI3Ks; these reactions are counteracted by the phosphatases inositol polyphosphate 4-phosphatase type II (INPP4B), SH2-containing inositol 5-phosphatase (SHIP-1/2) and phosphatase and tensin homolog (PTEN). Upon the activation of receptor tyrosine kinases (RTKs) or G protein-coupled receptors (GPCRs) on the plasma membrane, the class I PI3K isoforms (p110α, p110β, p110δ and p110γ) are recruited and convert PIP2 to PIP3 (Figure 1C) [10]. PIP3 binds to the pleckstrin homology (PH) domain of Akt, triggering conformational rearrangement and recruiting Akt to the plasma membrane [10,11]. This process facilitates the phosphorylation of Akt at the Thr308 and Ser473 residues, with PDK1 and mTORC2 acting as pivotal upstream kinases responsible for these modification events. Once fully activated, Akt detaches from the cell membrane and translocates to the cytoplasm or nucleus [12,13]. It subsequently activates mTORC1 by phosphorylating two negative regulatory factors of mTORC1, namely TSC2 and PRAS40. The activated mTORC1 further phosphorylates p70S6K and 4EBP1, ultimately regulating cell proliferation, metabolism, growth, survival, cell cycle progression, and protein synthesis [14,15]. Meanwhile, the lipid phosphatase PTEN negatively regulates this pathway by dephosphorylating PIP3 back to PIP2 (Figure 1C) [10]. PI3K-targeted inhibitors can mitigate adverse side effects induced by conventional chemotherapeutic agents [16].

To date, multiple PI3K inhibitors have been successfully developed for tumor-targeted therapy. Among them, alpelisib (a PI3Kα inhibitor) and inavolisib (aPI3Kα inhibitor) have been approved for marketing by the FDA to treat breast cancer, while CYH33 (a PI3Kα inhibitor) has obtained marketing approval from the Japanese Ministry of Health, Labor and Welfare for solid tumors [6]. Copanlisib, a pan-inhibitor against class I PI3K isoforms, is used to manage follicular lymphoma (FL) [6]. The PI3Kδ-selective agent idelalisib is recommended for a subset of B-cell cancers [7]. Duvelisib with dual activity toward PI3Kδ and PI3Kγ is available for chronic lymphocytic leukemia (CLL), FL and small lymphocytic lymphoma (SLL) [7]. Likewise, umbralisib (PI3Kδ inhibitor) is approved to treat patients with CLL, FL and marginal zone lymphoma (MZL) [7]. Although existing PI3K inhibitors have therapeutic effects in tumors, they face problems such as limited drug types, drug resistance, and so on [17,18]. These challenges necessitate the development of novel PI3K inhibitors with better pharmacological characteristics.

In our work, we discovered KTC101 with a strong binding ability to PI3K and good druggability through virtual screening. After chemical synthesis, we evaluated its kinase inhibitory activity and in vitro anti-tumor cell proliferation ability to maximize its potential in tumor treatment and provided data support for finding suitable indications. Beyond discovering a novel PI3K inhibitor candidate, this research also delivers new strategic insights for targeted tumor therapy.

## 2. Materials and Methods

### 2.1. In Silico Screening Based on Molecular Docking

As a mature computational simulation technology, virtual screening is one of the core technical means in modern new drug development research. This study adopted the Discovery Studio 3.5 platform to perform virtual screening procedures on the basis of molecular docking simulations [19]. The ligand-binding domain of PI3Kδ (PDB ID: 2WXL) was accurately localized with the assistance of the “Define and Edit Binding Sites” function [20]. Following this step, binding pockets covering the key residues of PI3Kδ were established using the “From Current Selection” module. The key residues around the binding site included MET752, SER754, PRO758, TRP760, ILE777, LYS779, LEU784, ASP787, LEU791, TYR813, CYS815, ILE825, GLU826, VAL827, VAL828, SER831, THR833, MET900, PHE908, ILE910, ASP911 and PHE912. The binding site is a sphere (X = −9.31Å, Y = −30.75 Å, and Z = 23.75 Å) and the radius is 7.39 Å. This virtual screening was performed based on an in-house small-molecule library containing 1883 entries covering triazine derivatives, pyridine derivatives, phenols, flavonoids, anthraquinone and other structural types. In our virtual screening, the -CDOCKER energy was used as the primary filter to prioritize candidate compounds. Specifically, we retained compounds with -CDOCKER energy higher than 24.8970 kcal/mol. The preparation of the ligand was performed through the Prepare ligand module. The CDOCKER algorithm was implemented to further analyze the docking energy between the screened candidate compounds and PI3Kδ.

### 2.2. Assessment of Drug-like Properties

A great number of drugs fail to get through the clinical trial phase on account of inadequate pharmacokinetic parameters [21,22]. Therefore, it is essential to forecast the pharmacokinetic characteristics of compounds prior to their entry into clinical research. The structural features of drugs dictate their physicochemical and biochemical properties, which in turn determine their pharmacokinetic behaviors and toxicological effects. In this section, the ADMET algorithm module within Discovery Studio 3.5 and ADMET 3.0 software are employed to make predictions regarding the pharmacokinetics and toxicity of the target compounds [23,24]. The content of the evaluation is as follows: aqueous solubility, CYP2D6 binding, intestinal absorption, mouse female/male FDA, Ames prediction, hepatotoxic prediction, DTP prediction, skin sensitization and so on. Following the screening of candidate PI3Kα inhibitors in the foregoing experiments, ADMET lab 3.0 was adopted to further verify the druggability of these compounds, including topological polar surface area (TPSA), number of rings (nRing), and so on.

### 2.3. Molecular Dynamics Simulations

Molecular dynamics (MD) simulations were performed using GROMACS 2020.3 software. The amber99sb-ildn force field and the general Amber force field (GAFF) were used to generate the parameters and topologies of proteins and ligands, respectively. The size of the simulation box was set so that the distance between each atom of the protein and the box boundary was greater than 1.0 nm. The box was filled with an explicit SPC216 water model, and Na^+^ and Cl^−^ counterions were added to neutralize the simulation system. The entire system was optimized by the steepest descent method to eliminate unfavorable contacts and atom overlaps. To achieve sufficient pre-equilibration of the simulation system, the NVT ensemble and the NPT ensemble were performed for 100 ps at 300 K and 1 bar, respectively. The MD simulation of 100 ns was performed with periodic boundary conditions, and the temperature (300 K) and pressure (1 bar) were controlled by the V-rescale and Parrinello–Rahman methods, respectively. The Newton equation of motion was calculated using the leapfrog integration with a time step of 2 fs. The long-range electrostatic interaction was calculated by the Particle Mesh Ewald (PME) method using Fourier spacing of 0.16 nm, and the LINCS method was used to constrain all bond lengths [25].

### 2.4. Prediction of Metabolic Pathways and Metabolites

BioTransformer 3.0 was utilized to analyze the metabolic pathways and metabolites of compound KTC101, so as to thoroughly explore the properties of this compound [26]. Specifically, the metabolic pathways chosen encompassed phase I (CYP450) and phase II transformations. Afterwards, the SDF file of compound KTC101 was uploaded, followed by clicking the submit button. Ultimately, the presented data were sorted out and subjected to analytical procedures.

### 2.5. Synthesis and Characterization

A Bruker AVANCE III 400 MHz spectrometer (Bruker BioSpin GmbH, Ettlingen, Germany) was utilized to obtain ^1^H and ^13^C NMR spectra. High-resolution electrospray ionization mass spectra (HRMS) were recorded on an Agilent 6224 ESI/TOF mass spectrometer (Agilent Technologies, Santa Clara, CA, USA). The purity of the compound was determined by HPLC using a Shimadzu Prominence system (Shimadzu Corporation, Kyoto, Japan) fitted with a Venusil XBP C18 column (5 μm, 150 Å, 4.6 × 250 mm, Agela Technologies, Tianjin, China). The mobile phase consisted of (A) water containing 0.1% formic acid and (B) methanol, and the flow rate was 1 mL/min. The isocratic elution program was 5:95 (A:B) for 15 min; the gradient elution program was 95:5 → 5:95 (A:B) over 0–10 min, followed by 5:95 (A:B) over 10–25 min.

#### 2.5.1. Synthesis of 4-(4,6-Dichloro-1,3,5-triazin-2-yl)morpholine

2,4,6-trichloro-1,3,5-triazine (1.0 g, 5.4 mmol) was dissolved in acetone (1 mL) and cooled to −20 °C. A solution of morpholine (0.3 g, 3.4 mmol) and triethylamine (0.4 g, 3.9 mmol) in acetone (10 mL) was then added, and the mixture was stirred at −20 °C for 30 min, during which the reaction progress was monitored by TLC. Upon completion, water was added to precipitate the product. The precipitate was collected by filtration, washed with water and acetone, and dried under vacuum to give a white solid (yield 59.7%). ^1^H NMR (400 MHz, CDCl_3_) *δ* 3.93–3.85 (m, 4H, CH_2_ of morpholine), 3.79–3.72 (m, 4H, CH_2_ of morpholine).

#### 2.5.2. Synthesis of 4-(4-Chloro-6-(2-(difluoromethyl)-1*H*-benzo[*d*]imidazol-1-yl)-1,3,5-triazin-2-yl)morpholine

A mixture of 2-difluoromethyl-1*H*-benzo[*d*]imidazole (0.42 g, 2.5 mmol), 4-(4,6-dichloro-1,3,5-triazin-2-yl)pyridine (0.59 g, 2.5 mmol), and K_2_CO_3_ (1.38 g, 10 mmol) in DMF (10 mL) was stirred at room temperature for 4 h, and the reaction progress was monitored by TLC. Upon completion, water (20 mL) was added to dissolve the inorganic salts, and the desired product precipitated as a white solid. The precipitate was collected by filtration, washed with water and acetone, and dried to give the product as a white solid in 38% yield. ^1^H NMR (400 MHz, CDCl_3_) *δ* 8.43 (d, *J* = 8.1 Hz, 1H, CH of benzene ring), 7.91 (d, *J* = 7.6 Hz, 1H, CH of benzene ring), 7.74–7.42 (m, 3H, CH of benzene ring and CHF_2_), 4.03–3.92 (m, 4H, CH_2_ of morpholine), 3.89–3.77 (m, 4H, CH_2_ of morpholine).

#### 2.5.3. Synthesis of 4-(4-(2-(Difluoromethyl)-1*H*-benzo[*d*]imidazol-1-yl)-6-morpholino-1,3,5-triazin-2-yl)-1,4-oxazepane

4-(4-chloro-6-(2-(difluoromethyl)-1*H*-benzo[*d*]imidazol-1-yl)-1,3,5-triazin-2-yl)morpholine (0.3 g, 0.8 mmol) was dissolved in homomorpholine (3 g, 29.7 mmol), and the mixture was stirred at 70 °C for 2 h, during which the reaction progress was monitored by TLC. Upon completion, the reaction mixture was diluted with water, and the resulting precipitate was collected by filtration, washed with water, and dried to give KTC101 as a white solid in 18% yield.

### 2.6. Adapta Kinase Assay

To study the PI3K inhibitory activity of KTC101, the kinase activity of PI3Ks in the presence or absence of the compounds was measured by the Adapta kinase assay. The PI3K kinase reaction was carried out in a 10 μL reaction system (384-well plate) containing diluted compounds, PI3Ks, 2 mM DTT, 0.1 mM substrate and 0.02 mM ATP, with 1 h of incubation at room temperature. After adding 5 μL of a solution of 30 mM EDTA, 6 nM antibody, and 12 nM tracer, the mixture was incubated for another 30 min. The remaining kinase activity was determined by the formula: kinase activity (% control) = (sample − minus enzyme control)/(plus enzyme control − minus enzyme control) × 100. Data were analyzed by the GraphPad Prism 5 software.

### 2.7. Cell Lines and Culture

We sourced HSC3 and HSC4 cell lines from the American Type Culture Collection (ATCC, Manassas, VA, USA). Each cell line was maintained in media specified (Wuhan Servicebio Technology Co., Ltd., Wuhan, China), ensuring optimal growth conditions. Specifically, cells were cultured in Minimum Essential Medium Eagle (MEM-E) supplemented with 10% fetal bovine serum (FBS) and 1% penicillin-streptomycin at 37 °C in an incubator containing 5% CO_2_. Both cell lines are adherent and were passaged when they reached 70–80% confluence and were used within passage 15.

### 2.8. Cell Proliferation Assay

Cell proliferation was performed by MTT assay as described previously according to the manufacture’s instructions [5]. HSC3 cells (6000 cells per well) and HSC4 cells (5000 cells per well) were seeded into 96-well plates at a volume of 200 μL per well. The two cell lines have intrinsic differences in proliferation rate and adhesion characteristics, leading to different cell-seeding densities. The seeding parameters used in this manuscript ensured that both cell lines were in the logarithmic growth phase at the experimental endpoint, and their OD values fell within the linear detection range. After 16 h, cells were treated with DMSO (final concentration of 0.25%, *v*/*v*, 0.5 μL) or a series of concentrations of KTC101 (0.5 μL). All compounds involved in the experiments were dissolved in DMSO. After a 48 h incubation period, 20 μL of MTT (5 mg/mL) was added to each well. After further incubation for 4 h at 37 °C, the absorbance at 490 nm was measured using an iMark microplate reader (BIO-RAD, Hercules, CA, USA). Three independent replicates were performed for each experimental point. Data were processed by Graphpad Prism 5 software to calculate IC_50_.

## 3. Results

### 3.1. Virtual Screening

**Figure 2:**
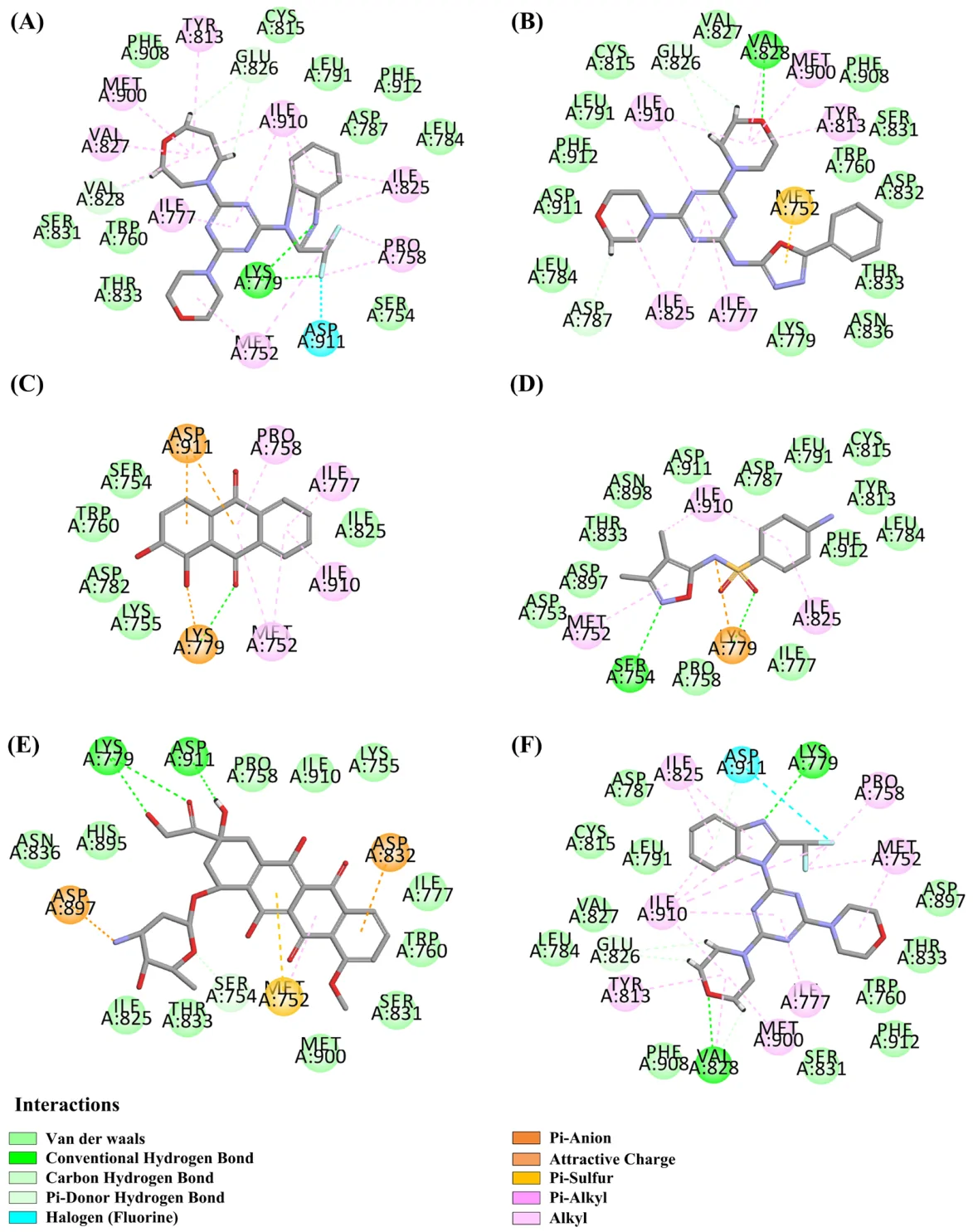
(**A**) KTC101-PI3Kδ binding modes. (**B**) H-60-PI3Kδ binding modes. (**C**) Alizarin-PI3Kδ binding modes. (**D**) Gantrisin-PI3Kδ binding modes. (**E**) Ellence-PI3Kδ binding modes. (**F**) ZSTK474-PI3Kδ binding modes.

Through virtual screening, we selected compounds that can form hydrogen bonds (H-bonds) with key amino acids in the target site and rank among the top eight in -CDOCKER energy. The chemical structures and corresponding -CDOCKER energies are listed in Table 1. The results showed that KTC101 had the highest -CDOCKER energy (36.5064 kcal/mol). KTC101’s benzimidazole group interacts with LYS779, and its homomorpholine group with VAL828, a key residue in the binding pocket, suggesting a strong inhibition mechanism similar to the action of ZSTK474 and the binding site (Figure 2A,F). Additionally, it can also form H-bonds with GLU826. The 3D binding mode diagram also revealed that KTC101 could bind to the ATP-binding pocket of PI3Kδ via the aforementioned interactions (Figure S1A,B). The docking result is similar to the binding mode of the original ligand ZSTK474. In addition, we also summarized the binding modes of several compounds with high docking scores to the target, among which H-60 can form H-bonds with VAL828, GLU826, and ASP787 (Figure 2B); Alizarin can form H-bonds with LYS779 (Figure 2C); Gantrisin can form H-bonds with SER754 and LYS779 (Figure 2D); and Ellence can form H-bonds with LYS779, SER754, and ASP911 in the binding site (Figure 2E).

### 3.2. Lipinski’s Filter and ADMET Study

**Figure 3:**
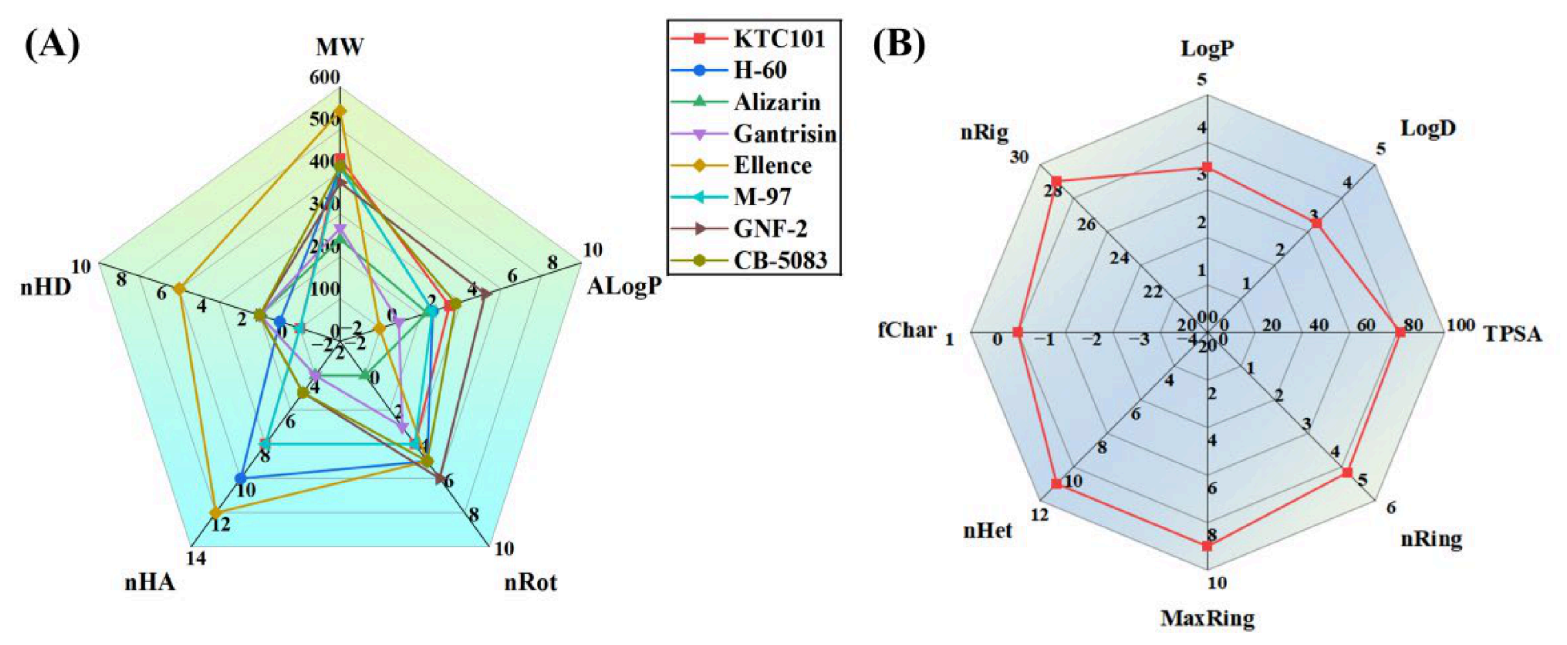
(**A**) Radar chart-based analysis of Lipinski’s rule of five for screened compounds. (**B**) Physicochemical property evaluation of KTC101.

Poor therapeutic efficacy and high toxicity are leading factors responsible for the failure of many drug candidates in research. Lipinski’s rule of five outlines critical molecular characteristics that determine the pharmacokinetic performance of drugs in the human body, covering MW, nHD, nHA, nRot, and AlogP. Here, we analyzed the physicochemical features and ADMET properties of the screened compounds to identify potential druggable novel PI3K inhibitors. According to the results in Figure 3A and Table S1, the vast majority of the tested molecules conformed to Lipinski’s rule of five, excluding Ellence.

The screened compounds were further assessed for multiple ADMET-related properties, including human intestinal absorption, water solubility, PPB, CYP2D6, PSA2D characteristics, rodent carcinogenicity, Ames mutagenicity, skin sensitization risk, hepatotoxicity, and developmental toxicity potential (DTP). All relevant evaluation outcomes are presented in Table 2 and Table 3. Human intestinal absorption and solubility are two key factors that affect oral bioavailability. The solubility levels of most compounds were 2 and 3, except GNF-2, indicating that the solubility of most compounds met the requirements (Table 2). According to the absorption scoring results, the eight screened molecules were graded 0, 1 and 3 for intestinal absorption, indicative of favorable moderate-to-high absorption profiles for most candidates excluding Ellence (Table 2). In clinical pharmacology, drug-induced CYP2D6 inhibitory effects are responsible for most drug–drug interaction events. From the experimental results, it can be seen that none of the compounds inhibit CYP2D6 (Table 2). In addition, the prediction results of toxicity (mouse male/female FDA, DTP prediction, Ames prediction, hepatotoxic toxicity, and skin sensitization) of eight molecules showed that KTC101, H60, and ZSTK474 had no risk of carcinogen, mutagenicity, DTP and skin sensitization (Table 3). However, during the hepatotoxicity assessment, it was found that H-60 carries a risk of hepatotoxicity (Table 3).

ADMET Lab 3.0 was utilized for further drug-likeness evaluation of KTC101, analyzing multiple molecular descriptors such as LogP, LogD, TPSA, nRing, MaxRing, nHet, fChar and nRig (Figure 3B). These properties have direct implications for the pharmacokinetic behavior and clinical potential of KTC101. First, the predicted LogP of 3.472 was within the optimal range (0–5) of Lipinski’s rule of five, indicating a favorable balance between aqueous solubility and membrane permeability, both of which are prerequisites for oral absorption and cellular penetration. Second, the TPSA of KTC101 (81.43 Å^2^) was well below the 140 Å^2^ threshold, which is associated with good passive intestinal absorption and oral bioavailability. Third, the favorable heteroatom content (nHet = 11) contributes to adequate aqueous solubility, and the electronically neutral charge state (fChar = 0) is favorable for passive membrane permeation. Finally, the ring number (nRing = 5), number of rigid bonds (nRig = 29), and number of atoms in the biggest ring (MaxRing = 9) all remained within their optimal ranges. Collectively, these results indicate that KTC101 possesses a favorable drug-like physicochemical profile, suggesting the potential for good oral absorption and bioavailability, and underscoring its clinical relevance.

These findings suggest that KTC101 meets the basic physicochemical requirements for good druggability. According to the virtual screening results and comprehensive analyses of Lipinski’s rule and ADMET characteristics, KTC101 was identified as the optimal lead compound for further systematic exploration.

### 3.3. Molecular Dynamics Simulation of KTC101-PI3Kδ System

**Figure 4:**
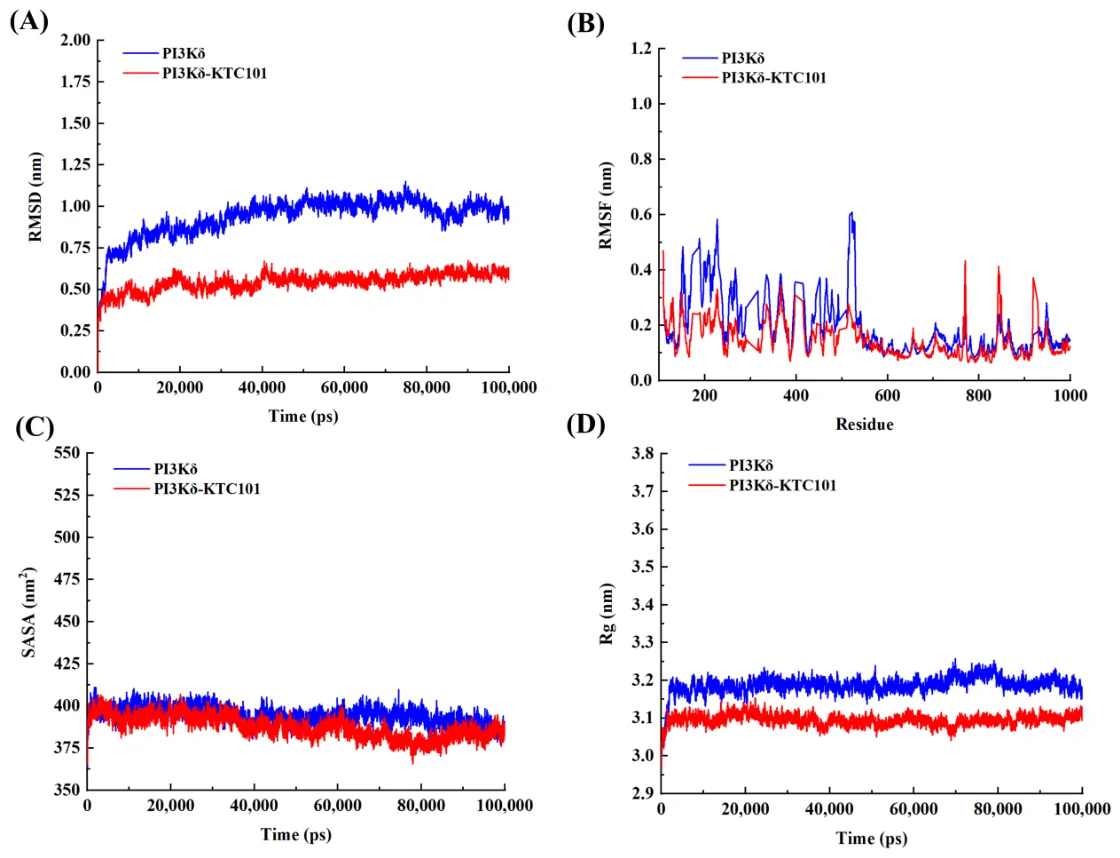
(**A**) The RMSD of KTC101-PI3Kδ/PI3Kδ. (**B**) The RMSF of KTC101-PI3Kδ/PI3Kδ. (**C**) Dynamic changes in SASA during simulations. (**D**) Variations in Rg during simulations.

As an essential evaluation parameter for molecular simulation stability, RMSD is applied to calculate atomic positional deviations from the initial reference structure. The steady state of RMSD curves suggests that the simulation system reaches equilibrium [27]. As illustrated in Figure 4A, KTC101-PI3Kδ and PI3Kδ stabilized after 20 ns of simulation, supporting the credibility of the simulation results. The lower RMSD of 0.561 nm for KTC101-PI3Kδ, compared with 0.980 nm for PI3Kδ, reflected its superior structural stability.

The root-mean-square fluctuation (RMSF) quantifies the deviation of individual atoms from their mean positions over time, reflecting the local flexibility of different protein regions [28]. As shown in Figure 4B, the RMSF values for KTC101-PI3Kδ and PI3Kδ were 0.145 nm and 0.191 nm, respectively. The lower RMSF observed in the KTC101-PI3Kδ system suggests that KTC101 enhances the structural stability of PI3Kδ.

Solvent-accessible surface area (SASA) is a vital parameter for evaluating the degree of surface exposure of proteins to surrounding solvents, and lower SASA values generally reflect better structural stability of the system [29]. Notably, the SASA values of PI3Kδ in both the KTC101 combination group and the free protein group gradually decreased throughout the simulation, as shown in Figure 4C. The average SASA values were 386.097 nm^2^ for the KTC101-PI3Kδ system, and 393.544 nm^2^ for the PI3Kδ system, respectively. The KTC101-PI3Kδ complex displayed the minimum SASA, reflecting enhanced structural stability and corroborating the RMSF findings.

The radius of gyration (Rg) can be utilized to characterize the compactness degree of protein spatial structure [30]. Lower Rg values correspond to a more compact protein conformation. As shown in Figure 4D, the KTC101-PI3Kδ (3.094 nm) exhibited greater structural compactness than the PI3Kδ (3.192 nm). Combined with the lower RMSF and SASA profiles obtained in prior analyses, the decreased Rg values further confirm the stable structural conformation of the KTC101-PI3Kδ complex.

**Figure 5:**
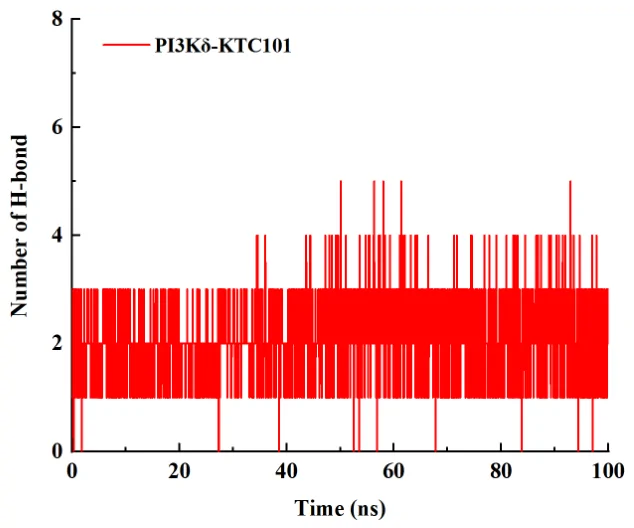
The number of H-bonds.

An H-bond analysis of simulation trajectories was performed to explore the intermolecular interactions between the protein and the ligand. Post-equilibrium statistical analysis revealed that the average H-bond number of the KTC101-PI3Kδ complex reached 2.09 (Figure 5), which solidly demonstrated its stable H-bond interaction characteristics.

We adopted the MMPBSA approach to dissect the binding energy of the KTC101-PI3Kδ complex. The total binding energy was partitioned into electrostatic, van der Waals, polar solvation, and nonpolar solvation terms, and all data are listed in Table S2. The results revealed a binding free energy of −374.627 kJ/mol for the KTC101-PI3Kδ system. The negative binding free energy indicated that the formation of the KTC101-PI3Kδ complex was thermodynamically favorable under the simulation conditions.

The binding mode and conformational stability of the KTC101–PI3Kδ complex during the MD simulation were further evaluated. As shown in Figure S2A, the 2D interaction diagram at the end of the simulation revealed that the complex was stabilized by multiple key interactions. In addition to forming H-bonds with GLU826 and VAL828 in the binding pocket, KTC101 also formed H-bonds with SER754, TYR813, VAL827 and ASP911. Moreover, the superimposed structures of the protein–ligand complex at 0 ns and 100 ns displayed a high degree of overlap (Figure S2B), indicating that the complex maintained a stable conformation throughout the 100 ns simulation. These findings were consistent with the RMSD, RMSF, SASA, Rg and H-bond analyses, collectively demonstrating the structural stability of the KTC101-PI3Kδ complex.

### 3.4. Analysis of Metabolic Pathways and Metabolites of KTC101

**Figure 6:**
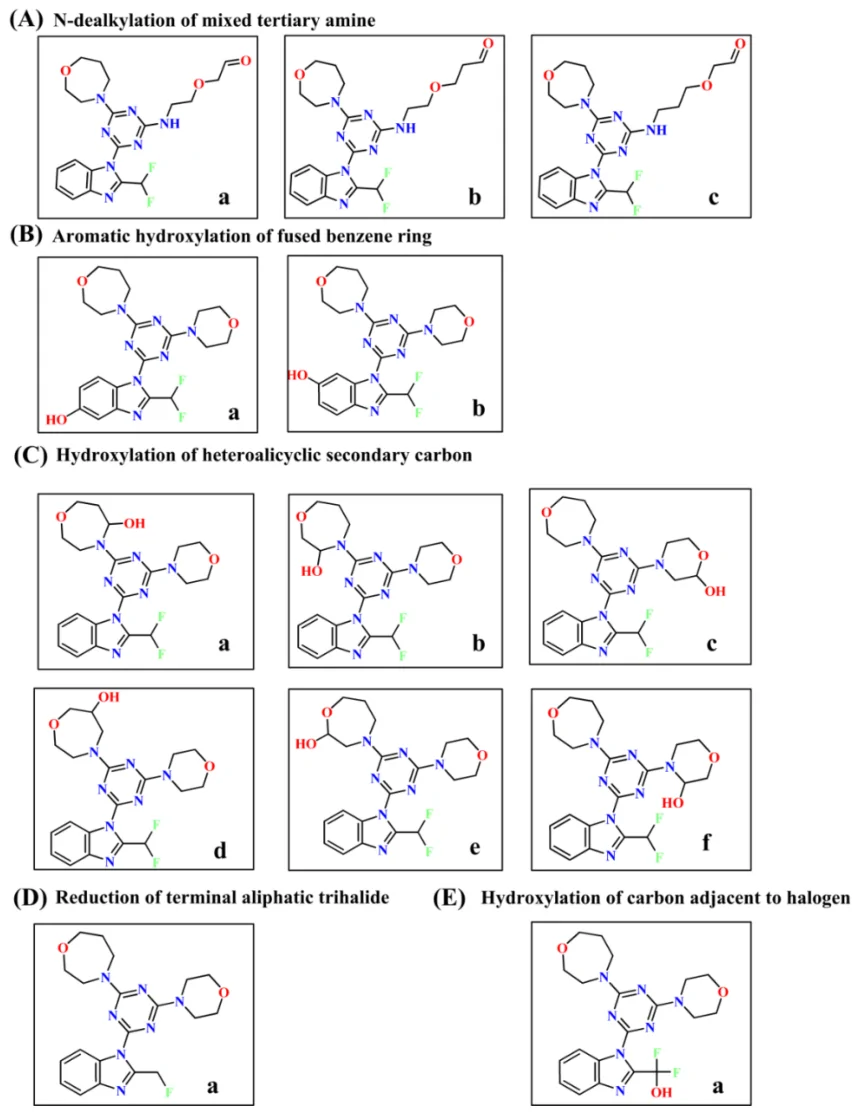
Metabolites and metabolic pathways of KTC101. (**A**) N-dealkylation of mixed tertiary amine and metabolites a–c. (**B**) Aromatic hydroxylation of fused benzene ring and metabolites a and b. (**C**) Hydroxylation of heteroalicyclic secondary carbon and metabolites a–f. (**D**) Reduction of terminal aliphatic trihalide and metabolite a. (**E**) Hydroxylation of carbon adjacent to halogen and metabolite a.

This study provides the first report of KTC101’s metabolic profile, demonstrating predominant hepatic transformation via phase I (CYP450) transformation, as summarized in Figure 6. KTC101 is metabolized through liver phase I metabolism, including the N-dealkylation of mixed tertiary amine, aromatic hydroxylation of the fused benzene ring, hydroxylation of a heteroalicyclic secondary carbon, reduction of a terminal aliphatic trihalide, and hydroxylation of a carbon adjacent to halogen, and the products were generated.

In drug metabolism, the N-dealkylation of mixed tertiary amines is a crucial type of reaction. It mainly occurs in the liver and is catalyzed by cytochrome P450 enzymes to remove an alkyl group from the tertiary amine structure of drug molecules, converting them into secondary or primary amines, improving water solubility, and accelerating drug excretion. KTC101 can be metabolized into a-c as shown in Figure 6A through this reaction. KTC101 undergoes the aromatic hydroxylation of the fused benzene ring reaction, introducing hydroxyl groups onto the benzene ring and metabolizing into a-b as shown in Figure 6B. This metabolic pathway is one of the most important phase I metabolic reactions of aromatic drugs, and the introduction of hydroxyl groups increases the polarity of the drug. When a drug molecule contains nitrogen/oxygen heterocyclic structures (such as pyridine, pyrrolidine, morpholine, etc.), the secondary carbon atom (-CH_2_-) on the ring is oxidized by liver CYP450 enzymes through hydroxylation of heterolicyclic secondary carbon, and metabolized into a heterocyclic ring containing hydroxyl groups. KTC101 can generate polar increasing metabolites a-f through this metabolic pathway (Figure 6C). The reduction of a terminal aliphatic trihalide is an important phase I metabolism that reduces lipophilicity, promotes elimination, terminates activity, and decreases halogen-related toxicity. KTC101 can generate metabolite a through this metabolic pathway (Figure 6D). Hydroxylation of the carbon adjacent to a halogen can increase polarity and accelerate elimination. KTC101 can generate metabolite a through this metabolic pathway (Figure 6E).

### 3.5. Synthesis and Structural Characterization of KTC101

**Scheme 1:**
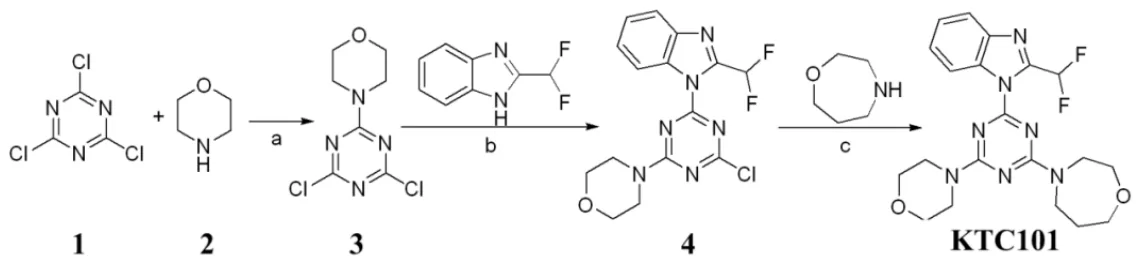
The synthetic route of KTC101. a: acetone, −20 °C; b: K_2_CO_3_, DMF, rt; c: 70 °C.

We successfully synthesized KTC101 through a series of chemical reactions (Scheme 1). The synthesis route of KTC101 used 2,4,6-trichloro-1,3,5-triazine (**1**) and morpholine (**2**) as starting materials to generate intermediate **3**. Intermediate **3** reacted with 2-(difluoromethyl)-1*H*-benzo[*d*]imidazole to form intermediate **4** under the catalytic action of K_2_CO_3_. Intermediate **4** and homomorpholine underwent substitution under heating conditions to obtain KTC101.

**Figure 7:**
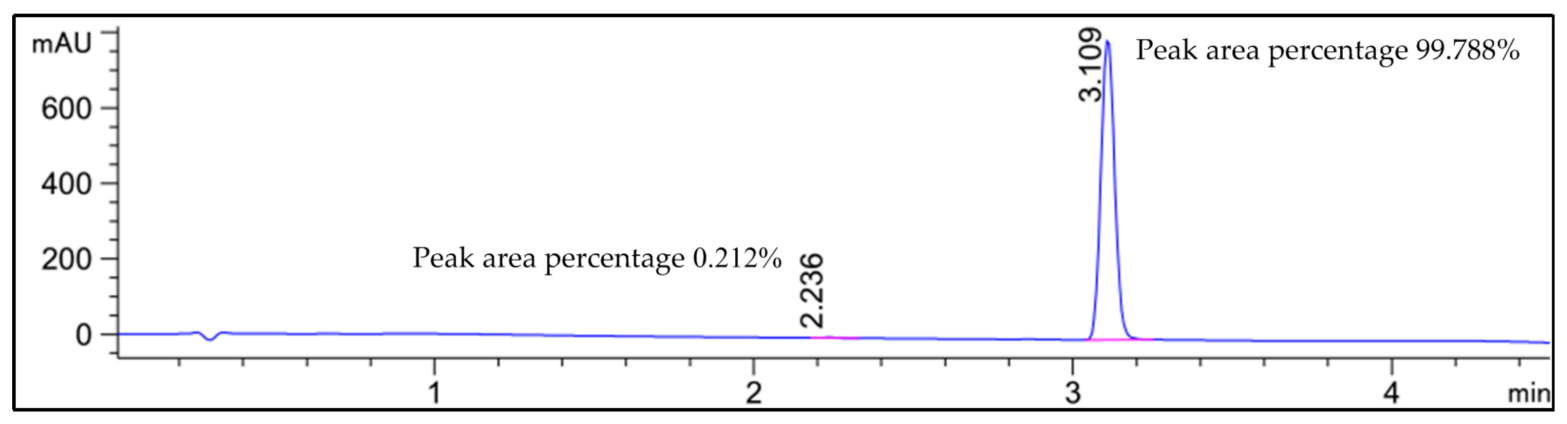
High-performance liquid chromatogram of KTC101.

**Figure 8:**
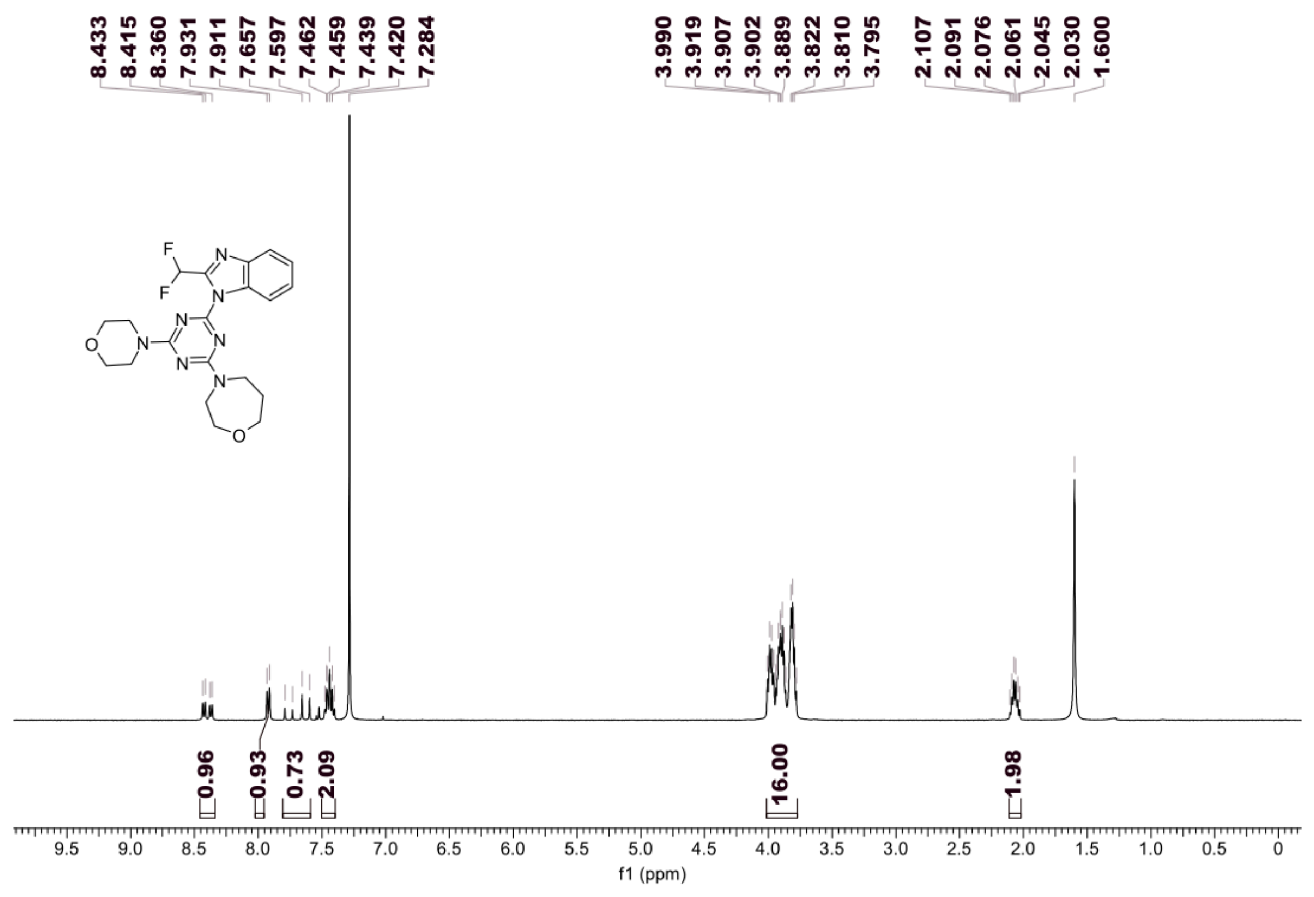
^1^H NMR Spectrum of KTC101.

**Figure 9:**
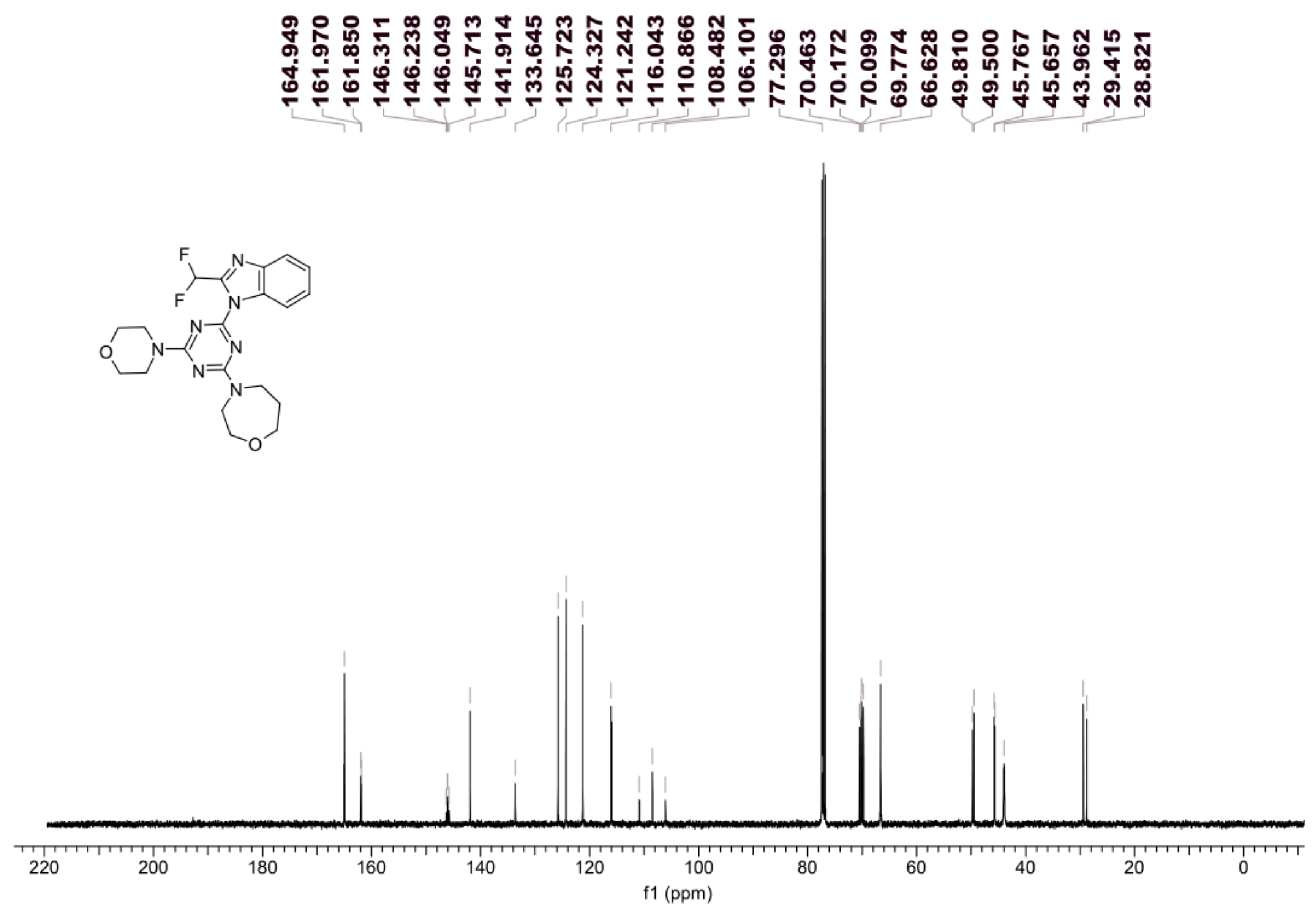
^13^C NMR Spectrum of KTC101.

**Figure 10:**
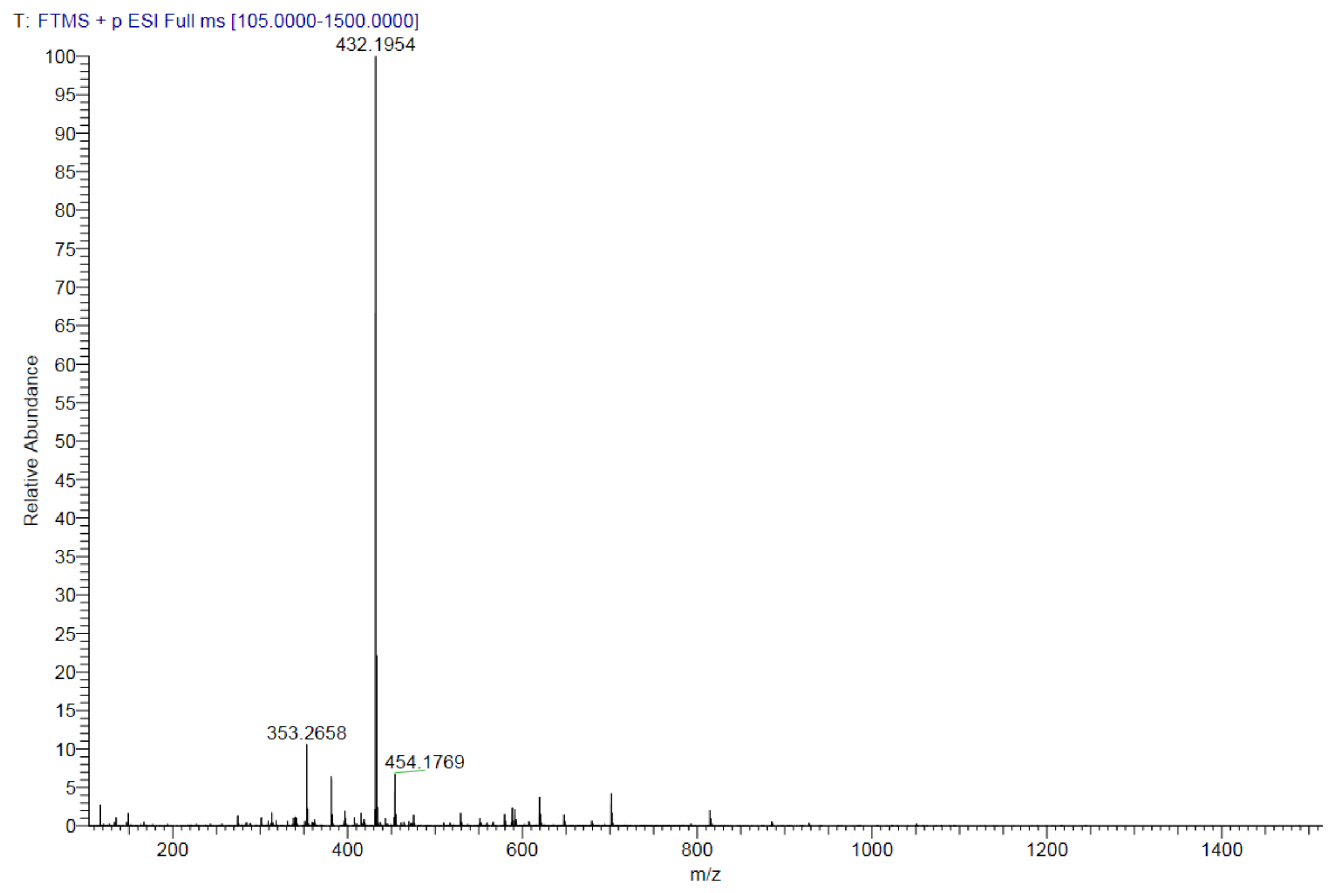
High-resolution mass spectrum of KTC101.

White solid with a yield of 18% and purity of 99.788% (Figure 7). Spectrum analysis: ^1^H NMR (400 MHz, CDCl_3_) *δ* 8.46–8.34 (m, 1H, CH of benzimidazole), 7.92 (d, *J* = 7.75 Hz, 1H, CH of benzimidazole), 7.81–7.59 (m, 1H, CH of benzimidazole), 7.50–7.40 (m, 2H, CH of benzimidazole and CHF_2_), 4.01–3.77 (m, 16H, CH_2_ of morpholine and 1,4-oxazepane), 2.07 (dq, *J* = 12.22, 6.14 Hz, 2H, CH_2_ of 1,4-oxazepane) (Figure 8). ^13^C NMR (100 MHz, CDCl_3_) *δ* 165.15–164.86 (m, 1C), 161.91 (d, *J* = 12.10 Hz, 1C), 146.01 (td, *J* = 26.41, 7.34 Hz, 1C), 141.91, 133.63 (d, *J* = 4.03 Hz, 1C), 125.72, 124.33, 121.24, 116.00 (d, *J* = 9.17 Hz, 1C), 108.47 (t, *J* = 238.0 Hz, 1C), 77.30, 70.46, 70.14 (d, *J* = 7.34 Hz, 1C), 69.77, 66.63, 49.81, 49.50, 45.71 (d, *J* = 11.00 Hz, 1C), 43.94 (d, *J* = 4.40 Hz, 1C), 29.42, 28.82 (Figure 9). HRMS (ESI): *m*/*z* calcd for C_20_H_24_F_2_N_7_O_2_ [M + H]^+^: 432.1960, found 432.1954 (Figure 10).

### 3.6. KTC101 Exhibits Potent Inhibitory Activity Against PI3K

**Figure 11:**
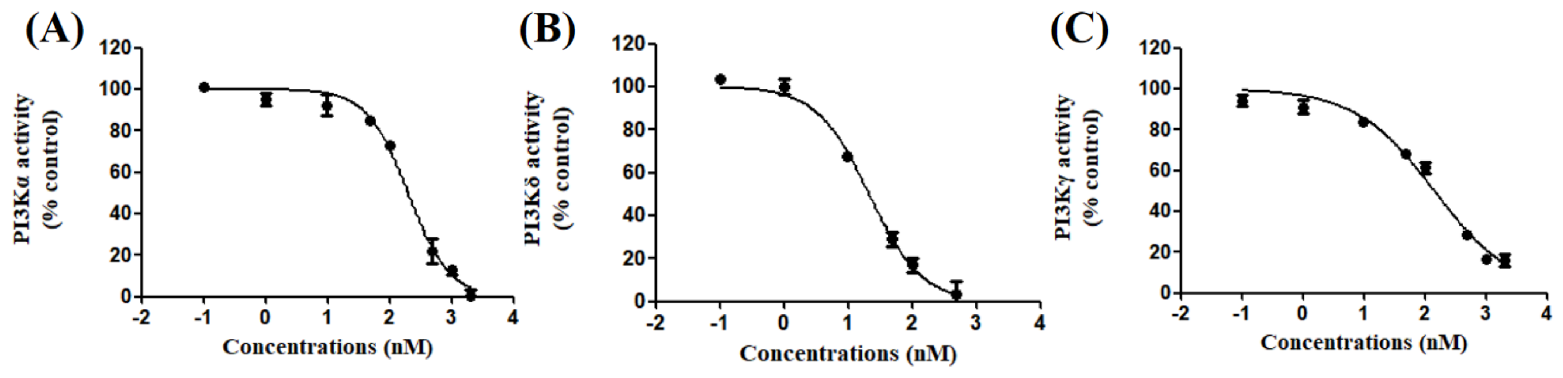
Inhibitory activity evaluation of KTC101 on human Class I PI3K isoforms ((**A**): PI3Kα; (**B**): PI3Kδ; (**C**): PI3Kγ). Data are presented as the mean ± SD (*n* = 3).

The inhibitory effect of KTC101 on the kinase activity of PI3K isoforms was evaluated using the Adapta kinase assay. As shown in Figure 11A–C and Figure S3A–C, KTC101 inhibited the activity of PI3Kα, PI3Kδ and PI3Kγ in a concentration-dependent manner compared with the DMSO-treated control. The half-maximal inhibitory concentration (IC_50_) values were 191.23 ± 40.84 nM, 23.30 ± 5.68 nM and 146.20 ± 21.82 nM for PI3Kα (Figure 11A), PI3Kδ (Figure 11B) and PI3Kγ (Figure 11C), respectively, indicating that the compound can significantly inhibit PI3K activity.

**Figure 12:**
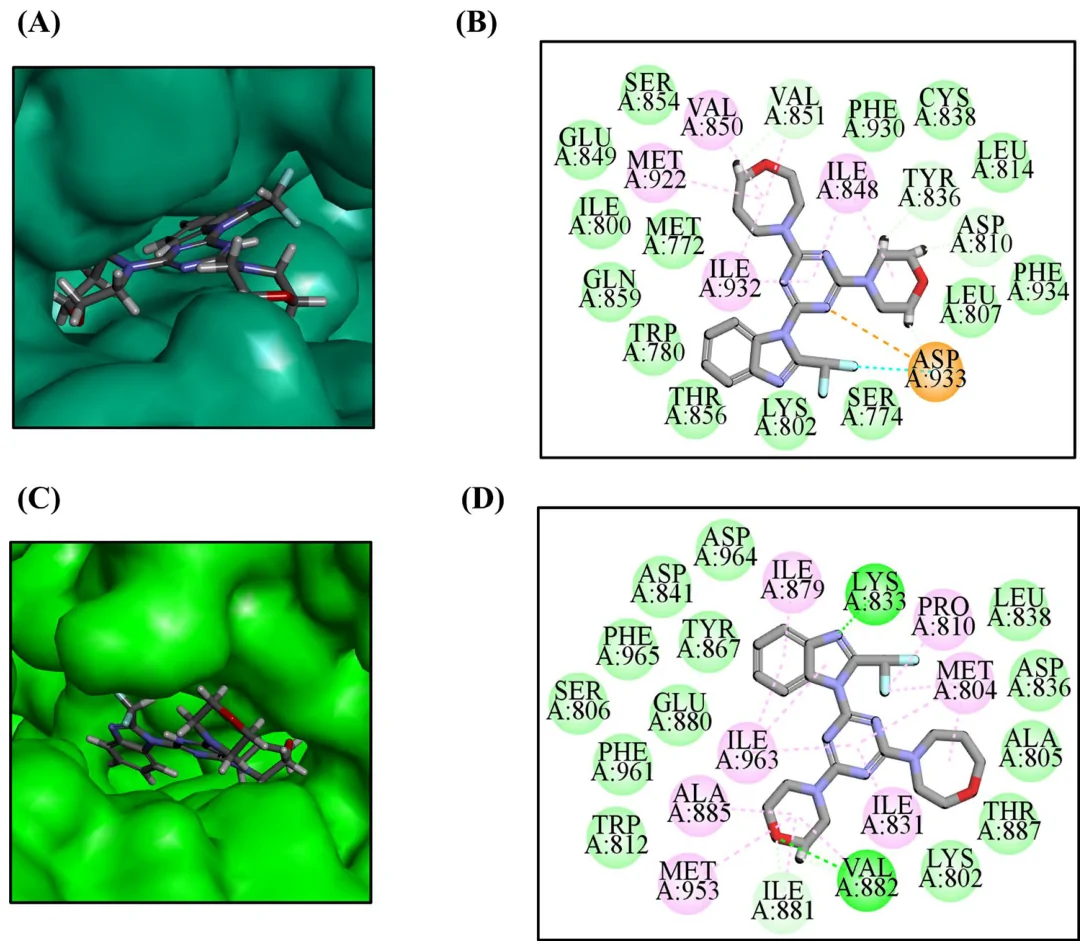
Analysis of the binding modes between KTC101 and PI3Kα/γ. (**A**) The binding pocket of PI3Kα inhibitors. (**B**) The binding modes between KTC101 and PI3Kα. (**C**) The binding pocket of PI3Kγ inhibitors. (**D**) The binding modes between KTC101 and PI3Kγ.

Molecular docking studies provided insights into KTC101’s interaction within the ATP binding pockets of PI3Ks (Figure 12): (1) For PI3Kα, the morpholine group of KTC101 forms key H-bonds with TYR836, and ASP810, while its homomorpholine interacts with VAL851. Interactions with the key hinge residue VAL851 are primarily responsible for its potent inhibitory activity against PI3Kα (Figure 12A,B). (2) For PI3Kγ, the benzimidazole group forms H-bonds with LYS833 and the morpholine group forms H-bonds with VAL882 (Figure 12C,D). The above experimental results indicate that KTC101 is an effective PI3K inhibitor with the strongest selectivity towards PI3Kδ.

### 3.7. KTC101 Exerts Potent In Vitro Anti-Proliferative Effects on HNSCC Cell Lines

**Figure 13:**
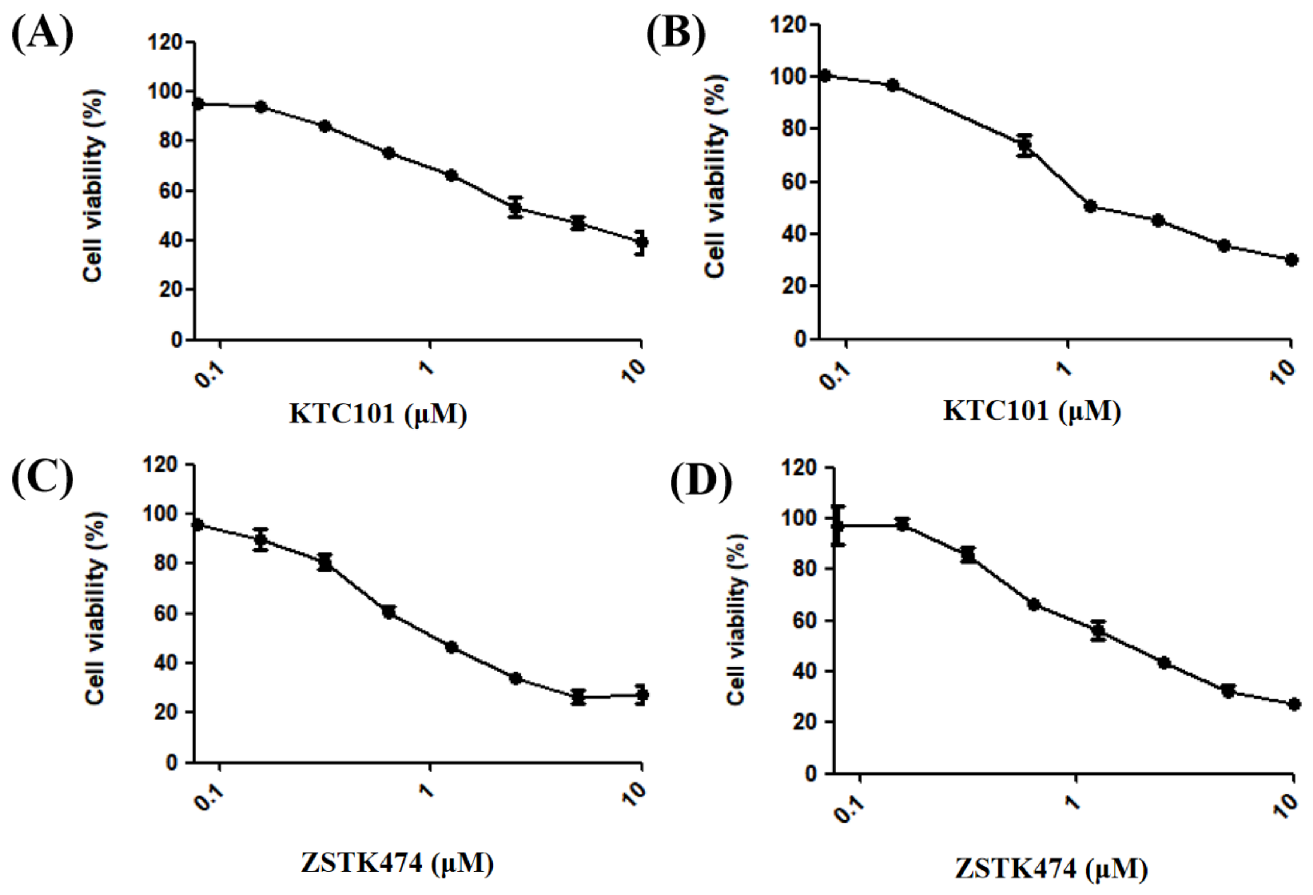
Cell viability assays illustrating the anti-proliferative effects of KTC101 on HSC3 cells (**A**) and HSC4 cells (**B**) following 48 h treatment with increasing concentrations. The anti-proliferative effects of ZSTK474 on HSC3 cells (**C**) and HSC4 cells (**D**) following 48 h treatment with increasing concentrations. Final DMSO concentration: 0.25%, *v*/*v*. Data are presented as the mean ± SD (*n* = 3).

The in vitro anti-proliferative activity of KTC101 and ZSTK474 was evaluated using the MTT assay. As shown in Figure 13A–D and Figure S4A–D, both compounds inhibited the viability of HSC3 and HSC4 cells in a concentration-dependent manner compared with the DMSO-treated control. The IC_50_ of KTC101 against HSC3 cells was 4.1 ± 0.73 μM (Figure 13A), and the IC_50_ for HSC4 cells was 2.2 ± 0.11 μM (Figure 13B). In addition, ZSTK474, a well-established PI3K inhibitor, exhibited an IC_50_ of 1.4 ± 0.17 μM toward HSC3 cells (Figure 13C) and 2.0 ± 0.10 μM for HSC4 cells (Figure 13D).

## 4. Conclusions

Given the critical involvement of PI3K in tumor onset and progression, extensive research has focused on developing small-molecule PI3K inhibitors for cancer therapeutic intervention. Over the past decade, numerous PI3K-targeted inhibitors have been synthesized, and several candidates have obtained clinical approval. Nevertheless, the clinical application of these early agents has achieved unsatisfactory therapeutic outcomes, which is mainly attributed to insufficient bioavailability, uncontrollable toxic side effects, and acquired drug resistance. Developing PI3K inhibitors with diverse structural scaffolds is the key to addressing the aforementioned limitations.

KTC101 represents a structurally novel PI3K inhibitor that markedly suppresses the kinase activity of various PI3K isoforms. It exhibits the strongest inhibitory potency against PI3Kδ (IC_50_ = 23.30 ± 5.68 nM) by forming H-bonds with key amino acid residues including LYS779, GLU826 and VAL828 within the active pocket of PI3Kδ. The homomorpholine moiety enables KTC101 to interact with GLU826 and VAL828 via H-bonds, improving its target selectivity. In addition, KTC101 exhibited IC_50_ values of 4.1 ± 0.73 μM and 2.2 ± 0.11 μM against HSC3 and HSC4 cells, respectively, which are comparable to those of the established PI3K inhibitor ZSTK474 (1.4 ± 0.17 μM and 2.0 ± 0.10 μM, respectively). KTC101 was considerably more potent than LY294002 in anti-proliferative activity against HSC4 cells. LY294002 required 25–50 μM to achieve significant growth inhibition in this cell line [31]. Furthermore, in HSC3 cells, KTC101 inhibited cell viability at concentrations within the same order of magnitude as those of LY294002 reported by Xu et al. [32]. Collectively, these findings corroborate that PI3K inhibition is an effective approach to suppress tumor cell growth and position KTC101 as a potent PI3K-targeted candidate worthy of further evaluation.

Although the present study preliminarily verified the tumor cell proliferation-inhibitory activity and kinase target selectivity of the tested compound, systematic safety evaluation at the cellular level remains insufficient. Further research will be carried out to supplement cytotoxicity tests on normal human cell lines and calculate the selective cytotoxicity index (SCI) to quantitatively characterize the compound’s tumor-selective killing effect. In addition, an in-depth mechanistic exploration will be performed, including cell-cycle distribution detection, apoptosis induction analysis and related signaling pathway verification, to clarify the intrinsic molecular mechanism underlying its anti-proliferative activity, which can provide more solid theoretical support for its subsequent anti-tumor development.

## Figures and Tables

**Table 1:** The structure characteristics and docking results of compounds.

Name	Structure	-CDOCKER Energy (kcal/mol)
KTC101	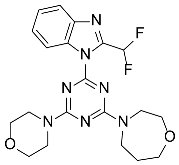	36.5064
H-60	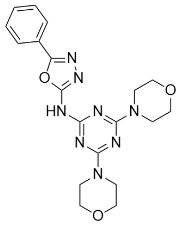	35.1304
Alizarin	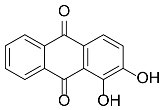	33.2523
Gantrisin	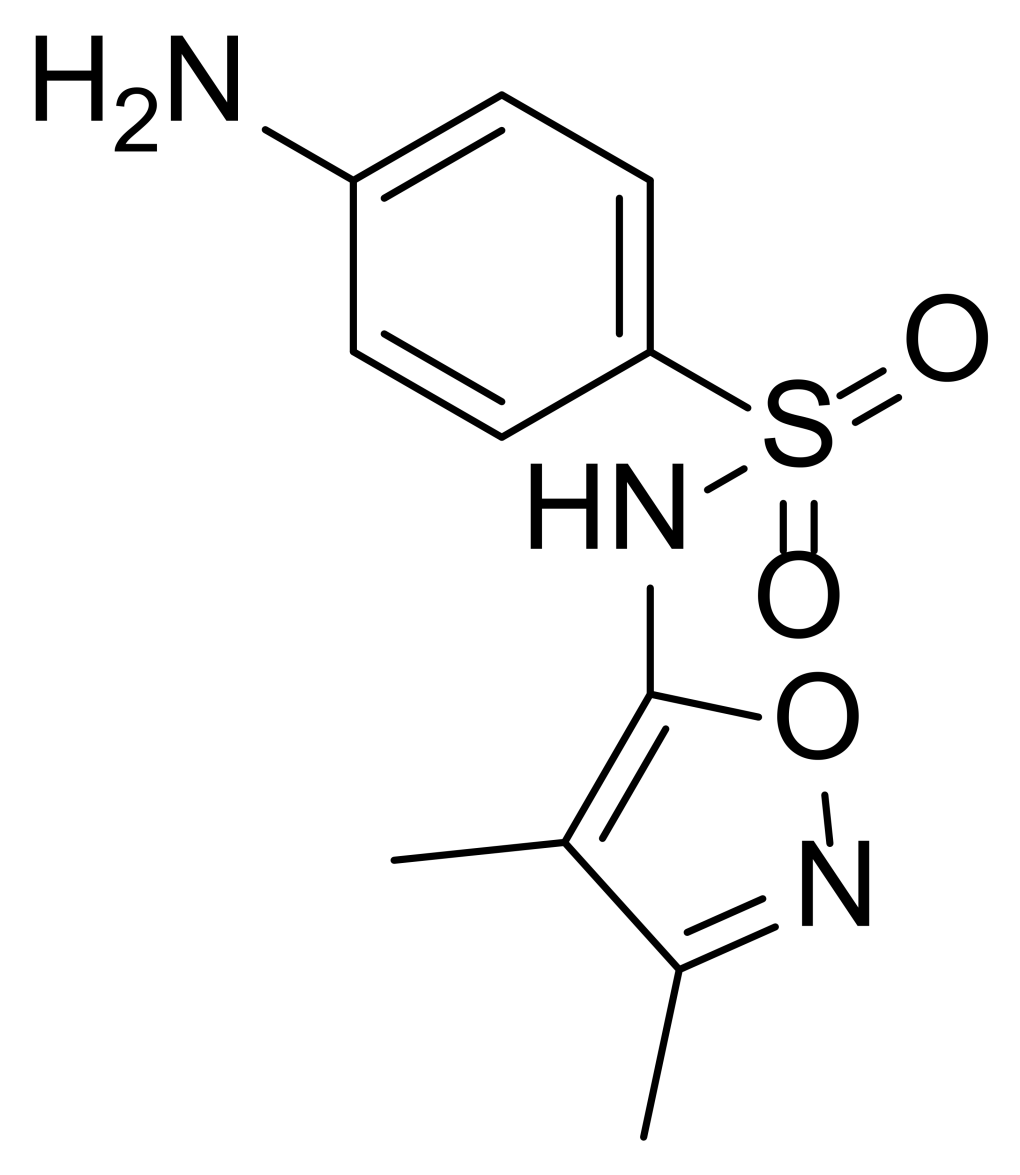	31.1850
Ellence	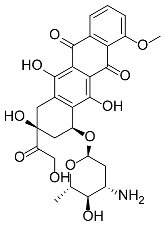	28.3526
M-97	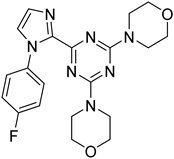	27.0812
GNF-2	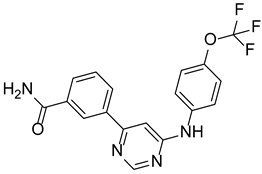	25.5660
CB-5083	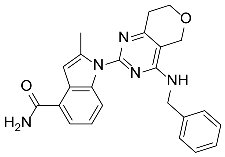	24.8970
ZSTK474	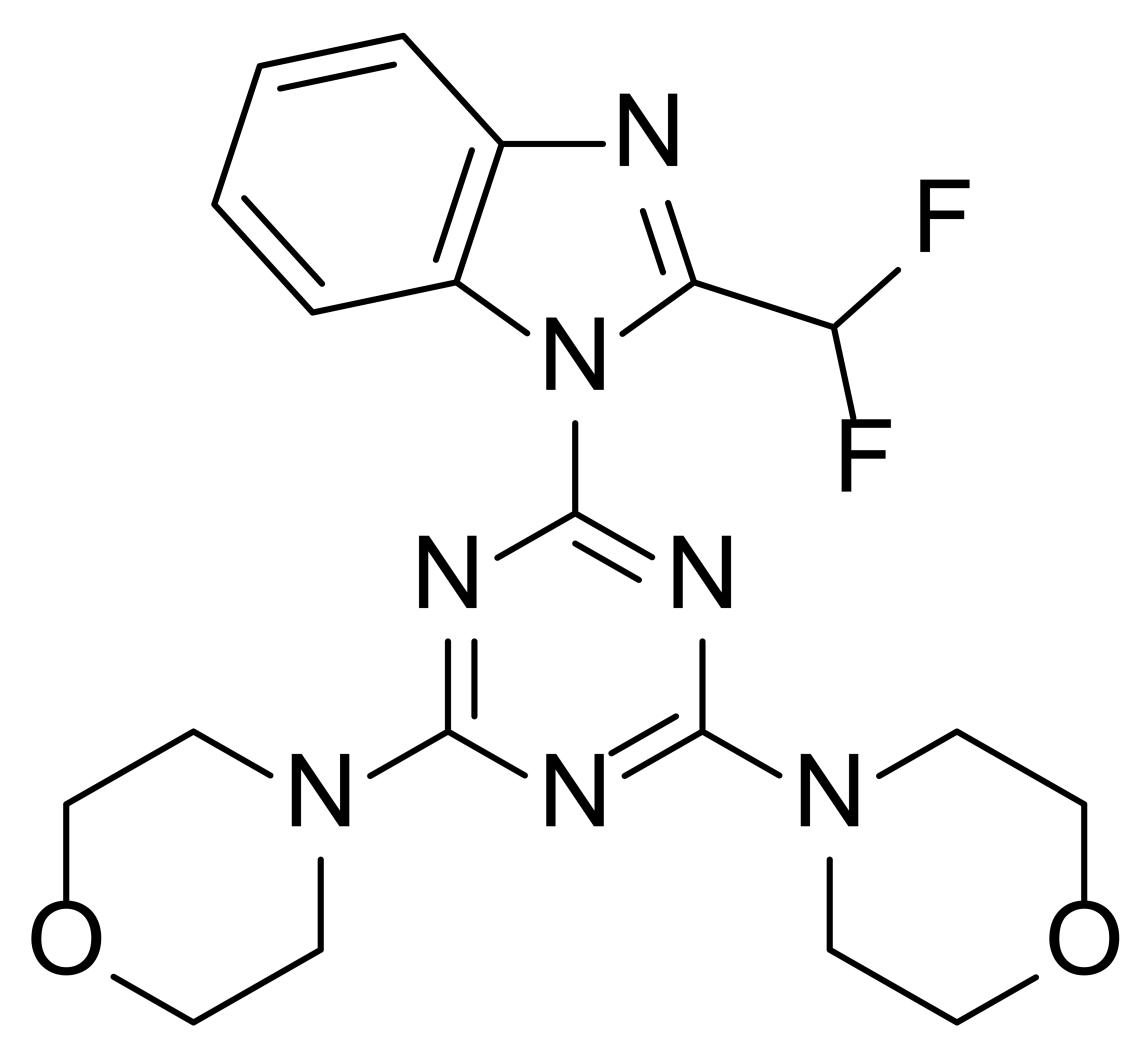	31.6225

**Table 2:** The ADME of the screened compounds.

ID Number	SolubilityLevel ^1^	CYP2D6 ^2^	AbsorptionLevel ^3^	PPB ^4^	PSA2D ^5^
KTC101	2	False	0	True	74.957
H-60	2	False	0	True	106.234
Alizarin	3	False	0	True	76.232
Gantrisin	3	False	0	True	97.767
Ellence	2	False	3	False	209.310
M-97	2	False	0	True	74.957
GNF-2	1	False	1	True	88.103
CB-5083	2	False	0	True	93.451
ZSTK474	2	False	0	True	74.957

1. Solubility_ Level: 1: very low, 2: yes, low, 3: yes, good, 4: optimal; 2. CYP2D6: False: <0.161, non-inhibitor, True: >0.161, inhibitor; 3. Absorption_ Level: 0: good, 1: moderate, 2: low, 3: very poor; 4. PPB: False: ≥90%, True: ≤90%; 5. PSA2D: ≤140.

**Table 3:** The TOPKAT prediction of the screened compounds.

ID Number	Mouse Female FDA	Mouse Male FDA	Ames Prediction	Hepatotoxic Prediction	DTP Prediction	Skin Sensitization
KTC101	Non-Carcinogen	Non-Carcinogen	Non-Mutagen	false	Non-Toxic	None
H-60	Non-Carcinogen	Non-Carcinogen	Non-Mutagen	true	Non-Toxic	None
Alizarin	Non-Carcinogen	Single-Carcinogen	Mutagen	true	Toxic	Strong
Gantrisin	Non-Carcinogen	Single-Carcinogen	Non-Mutagen	true	Non-Toxic	Strong
Ellence	Non-Carcinogen	Non-Carcinogen	Mutagen	true	Toxic	Weak
M-97	Multi-Carcinogen	Non-Carcinogen	Non-Mutagen	true	Non-Toxic	None
GNF-2	Single-Carcinogen	Single-Carcinogen	Mutagen	true	Non-Toxic	Strong
CB-5083	Non-Carcinogen	Non-Carcinogen	Non-Mutagen	true	Non-Toxic	Strong
ZSTK474	Non-Carcinogen	Non-Carcinogen	Non-Mutagen	false	Non-Toxic	None

DTP: developmental toxicity potential.

## Data Availability

The data presented in this study are available on request from the corresponding authors.

## Supplementary Materials

The following supporting information can be downloaded at: https://www.mdpi.com/article/10.3390/biology15191742/s1, Figure S1. (A) Three-dimensional (3D) binding mode of the KTC101-PI3Kδ complex. (B) The binding pocket of PI3Kδ occupied by KTC101. Grey stick: KTC101 and amino acid residue; Blue surface: protein. Figure S2. (A) Two-dimensional (2D) interaction diagram of the KTC101-PI3Kδ complex at the end of the 100 ns MD simulation. (B) Superimposed structures of the KTC101-PI3Kδ complex at 0 ns (yellow) and 100 ns (blue) of the MD simulation. Figure S3. The kinase activity of PI3Kα (A), PI3Kδ (B) and PI3Kγ (C) was measured after treatment with DMSO (solvent control) or increasing concentrations of the compound. Data are expressed as the percentage relative to the DMSO-treated group and presented as the mean ± SD (*n* = 3). * *p* < 0.05, ** *p* < 0.01, *** *p* < 0.001. Figure S4. Cell viability of KTC101 in HSC3 cells (A), HSC4 cells (B) and ZSTK474 in HSC3 cells (C) and HSC4 (D) cells by MTT assay. Cells were treated with DMSO (solvent control) or increasing concentrations of the KTC101 for 48 h. Data are presented as the mean ± SD *(n* = 3).* *p* < 0.05, ** *p* < 0.01, *** *p* < 0.001 versus the DMSO-treated group. Table S1. The Lipinski’s rule of five of the screened compounds. Table S2. The binding free energy (kJ/mol) of PI3Kδ with KTC101, and its components between receptor and ligand.

## Abbreviations

The following abbreviations are used in this manuscript:

PI3K	Phosphatidylinositol 3-kinase
HNSCC	Head and neck squamous cell carcinoma
PIP2	Phosphatidylinositol-4,5-bisphosphate
PIP3	Phosphatidylinositol-3,4,5-trisphosphate
pH	Pleckstrin homology
CLL	Chronic lymphocytic leukemia
FL	Follicular lymphoma
SLL	Small lymphocytic lymphoma
MZL	Marginal zone lymphoma
ABD	Adaptor binding domain
RBD	Ras binding domain
SH3	Src homology 3
BH	BCR-homology
N-SH2/i-SH2/C-SH2	N-terminal/intermediate/C-terminal Src-homology 2
PI	Phosphatidylinositol
PI3P	Phosphatidylinositol 3-phosphate
PI4P	Phosphatidylinositol 4-phosphate
PI4,5P_2_	Phosphatidylinositol 4,5-bisphosphate
PI3,4P_2_	Phosphatidylinositol 3,4-bisphosphate
PI3,4,5P_3_	Phosphatidylinositol 3,4,5-trisphosphate
PIP2	Phosphatidylinositol 4,5-bisphosphate
PIP3	Phosphatidylinositol 3,4,5-trisphosphate
INPP4B	Inositol polyphosphate 4-phosphatase type II
SHIP	SH2-containing inositol 5-phosphatase
PTEN	Phosphatase and tensin homolog
RTK	Receptor tyrosine kinase
GPCR	G protein-coupled receptor
PDK1	3-phosphoinositide-dependent protein kinase 1
mTORC1/2	Mechanistic target of rapamycin complex 1/2
p70S6K	p70 ribosomal S6 kinase
TSC2	Tuberous sclerosis complex 2
PRAS40	Proline-rich Akt substrate of 40 kDa
4EBP1	Eukaryotic translation initiation factor 4E-binding protein 1
MW	Molecular weight
nHD	Number of hydrogen bond donors.
nHA	Number of hydrogen bond acceptors.
nRot	Number of rotatable bonds
AlogP	Octanol–water partition coefficient
PPB	Plasma protein binding
PSA2D	2D polar surface area
DTP	Developmental toxicity potential
LogP	The logarithm of the n-octanol/water distribution coefficients
LogD	The logarithm of the n-octanol/water distribution coefficient at pH = 7.4
nRing	Number of rings
MaxRing	Number of atoms in the biggest ring
nHet	Number of heteroatoms
fChar	Formal charge
nRig	Number of rigid bonds
H-bonds	Hydrogen bonds
RMSD	Root mean square deviation
RMSF	Root mean square fluctuation
SASA	Solvent-accessible surface area
Rg	Radius of gyration
IC50	Half-maximal inhibitory concentration
