# Supplementary material for: In Silico Evaluation via Virtual Screening, ADMET Profiling and Molecular Dynamics Simulations: Synthesis and Biological Activity Analysis of a Novel Antitumor PI3K Inhibitor
Source: Biology (Basel). 2026 Oct 1;15(19):1742. doi: 10.3390/biology15191742 (PMC13663554; doi:10.3390/biology15191742)
Supplement: Supplementary file 1 [file biology-15-01742-s001.zip › biology-4485034-supplementary.pdf]

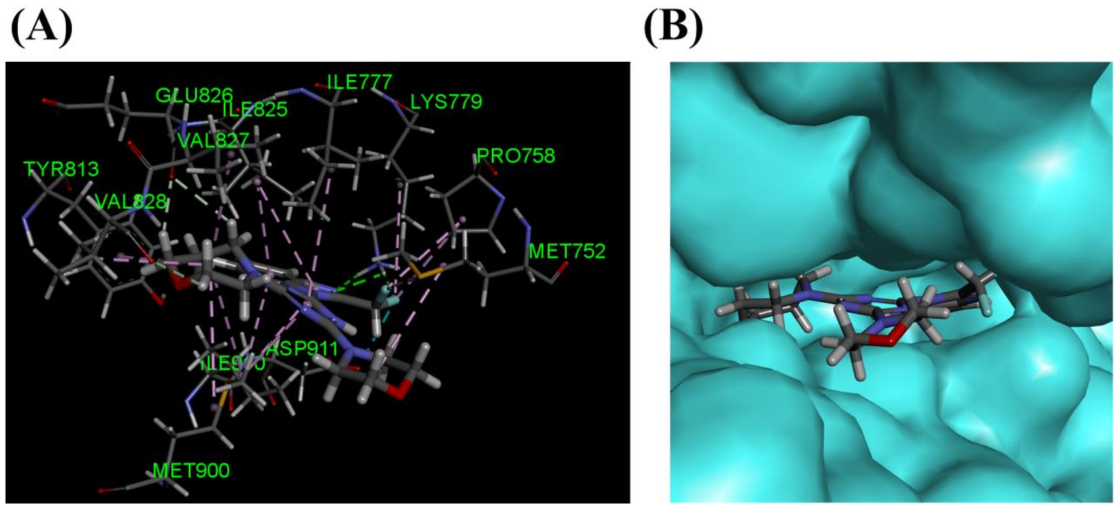

Figure S1. (A) Three-dimensional (3D) binding mode of the KTC101-PI3Kδ complex. (B) The binding pocket of PI3Kδ occupied by KTC101. Grey stick: KTC101 and amino acid residue; Blue surface: protein.

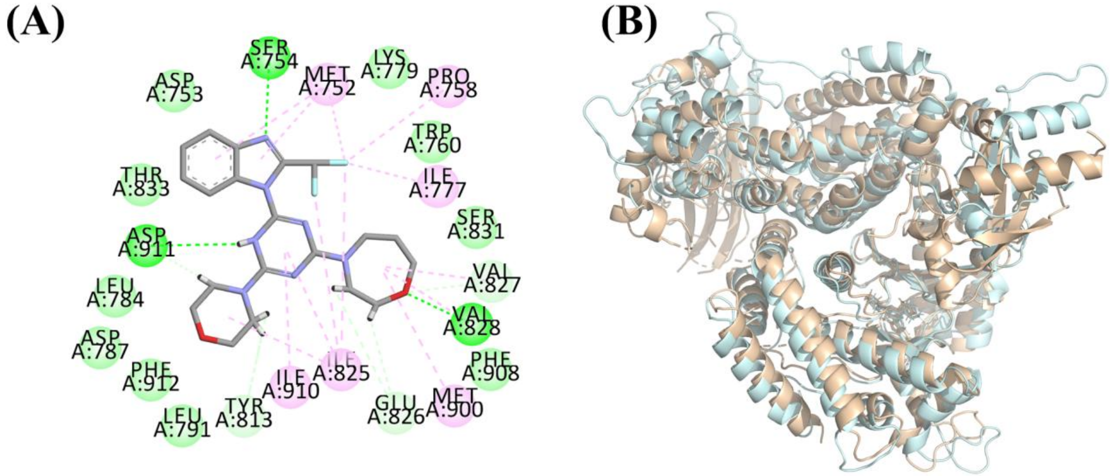

Figure S2. (A) Two-dimensional (2D) interaction diagram of the KTC101-PI3Kδ complex at the end of the 100 ns MD simulation. (B) Superimposed structures of the KTC101-PI3Kδ complex at 0 ns (yellow) and 100 ns (blue) of the MD simulation.

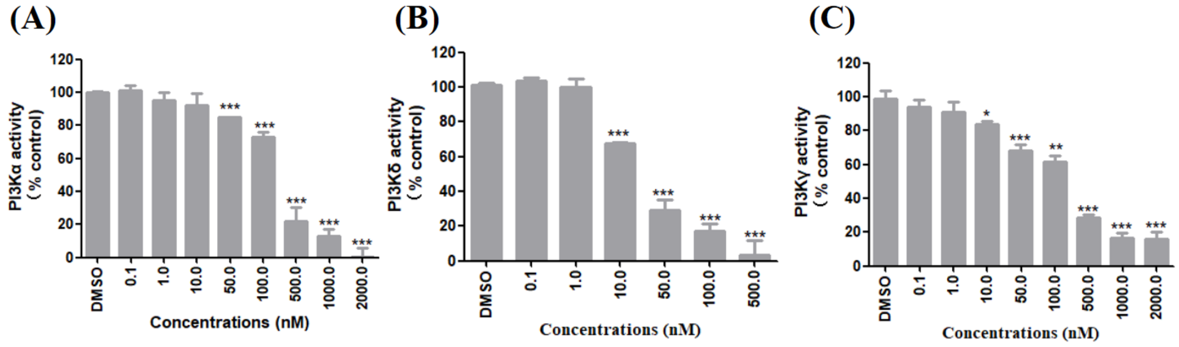

Figure S3. The kinase activity of PI3Kα (A), PI3Kδ (B) and PI3Kγ (C) was measured after treatment with DMSO (solvent control) or increasing concentrations of the compound. Data are expressed as the percentage relative to the DMSO-treated group and presented as the mean ± SD (n = 3). *$p < 0.05$, **$p < 0.01$, ***$p < 0.001$.

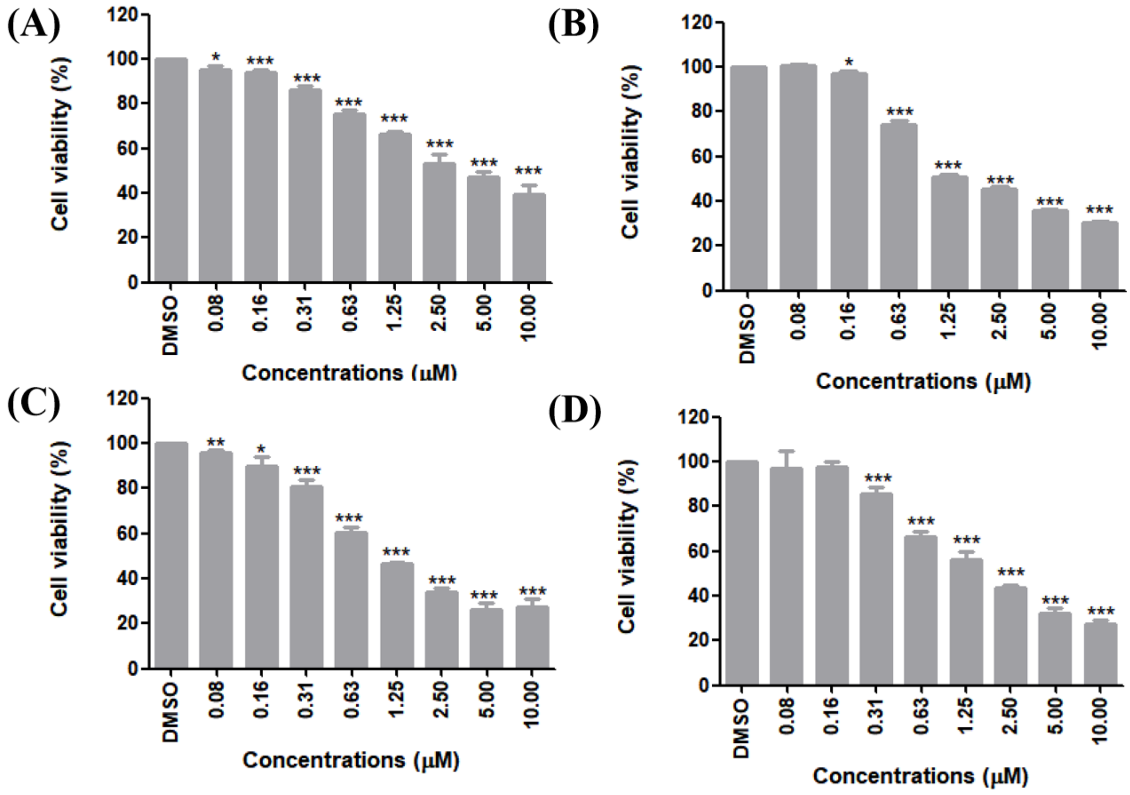

Figure S4. Cell viability of KTC101 in HSC3 cells (A), HSC4 cells (B) and ZSTK474 in HSC3 cells (C) and HSC4 (D) cells by MTT assay. Cells were treated with DMSO (solvent control) or increasing concentrations of the KTC101 for 48 h. Data are presented as the mean ± SD (n = 3).*$p < 0.05$, **$p < 0.01$, ***$p < 0.001$ versus the DMSO-treated group.

Table S1. The Lipinski's rule of five of the screened compounds.

ID number	MW[1]	ALogP[2]	nRot[3]	nHA[4]	nHD[5]
KTC101	431.439	3.429	4	8	0
H-60	410.430	2.599	5	10	1
Alizarin	240.211	2.324	0	4	2
Gantrisin	267.304	0.918	3	4	2
Ellence	543.519	-0.044	5	12	6
M-97	411.433	2.589	4	8	0
GNF-2	374.317	5.209	6	5	2
CB-5083	413.472	3.748	5	5	2

1. MW: 0~500; 2. ALogP: -2~6.5; 3. nRot: 0~11; 4. nHA: 0~10; 5. nHD: 0~5.

Table S2. The binding free energy (kJ/mol) of PI3Kδ with KTC101, and its components between receptor and ligand.

Energy	PI3Kδ-KTC101
Van der Waals energy (kJ/mol)	-216.852
Electrostatic energy (kJ/mol)	-400.837
Polar solvation energy (kJ/mol)	251.767
Nonpolar solvation energy (kJ/mol)	-24.124
Total binding energy (kJ/mol)	-390.046
TΔS (kJ/mol)	15.419
Total binding free energy (kJ/mol)	-374.627
